# Sensorimotor strategy selection under time constraints in the presence of two motor targets with different values

**DOI:** 10.1038/s41598-021-01584-w

**Published:** 2021-11-15

**Authors:** Ryoji Onagawa, Kazutoshi Kudo

**Affiliations:** 1 Laboratory of Sports Sciences, Department of Life Sciences, Graduate School of Arts and Sciences, The University of Tokyo, Tokyo, Japan; 2 Japan Society for the Promotion of Science, Tokyo, Japan; 3 Faculty of Science and Engineering, Waseda University, Tokyo, Japan

**Keywords:** Human behaviour, Decision, Motor control

## Abstract

Goal-directed movements often require choosing an option from multiple potential goals under time constraints. However, there are limited studies on how humans change their time spent on decision-making and movement patterns according to time constraints. Here, we examined how sensorimotor strategies are selected under time constraints when the target values are uncertain. In the double-target condition, the values were uncertain until the movement onset and presented immediately afterwards. The behavior in this condition was compared to the single-target condition, in relation to time constraints and target-separation-angles. The results showed that the participants frequently used the choice-reaction even under tight time constraints, and their performance was consistently lower than that in the single-target condition. Additionally, in the double-target condition, differences in the movement trajectory depending on the time constraint and target-separation angle were confirmed. Specifically, the longer the time constraint, the higher the frequency of the intermediate behavior (to initiate movement toward the intermediate direction of two targets) or the change-of-mind behavior (to change the aiming target during movement). Furthermore, the smaller the target-separation angle, the higher the frequency of intermediate behavior, but the frequency of change-of-mind was not affected by the target-separation angle. These results suggest that the participants initiated the movement at an incomplete value judgment stage in some trials. Furthermore, they seemed to select a strategy to utilize the information obtained during the movement, taking into account the time constraints and target-separation angle. Our results show a consistent cognitive bias in choosing a higher value when multiple alternatives have different values. Additionally, we also suggest flexibility and adaptability in the movement patterns in response to time constraints.

## Introduction

In many situations, decision making involves deciding on a single strategy among multiple action possibilities. In many studies of motor decision-making, the focus has been on how to output an action according to a prior gain function in advance^1–9^. On the other hand, in sports and other decision-making situations, it is necessary to collect information about the value of potential goals in a limited time while planning and executing the movement.

In such situations, to maximize the performance, strategy selection is important for the speed-accuracy trade-off^9–11^, such as the relationship between movement speed and movement variability^12,13^ or between decision speed and decision accuracy^10,14,15^. For choice behavior, in particular, the Hick–Hyman law^16,17^ describes the fundamental human property whereby reaction times for making decisions become longer as the number of options increases because having more options requires more time to accumulate information.

In a situation where there are two alternatives, for example, the response strategy can be divided into two categories: a simple reaction, in which an individual quickly responds to the predetermined choice, and a choice reaction, in which an individual responds by choosing the more rewarding of the two choices. The simple reaction strategy, while having temporal effectiveness, is disadvantageous in terms of the amount of information obtained. In contrast, the choice-reaction strategy allows us to choose better alternatives by increasing the amount of information available but at the cost of time. Therefore, in choosing such a strategy, the values of both time and amount of information should be considered.

In our previous study^10^, we used a selective-response task between two alternatives with progressively decreasing gains or probabilities of each option over time to test whether strategy optimization occurs in terms of reward maximization. By varying the rate of decrease in value from trial to trial and examining strategy switching, we found that although participants changed their response strategies according to the rate, there was a bias toward over-preferring the choice-reaction strategy in situations where the gain decreased over time. This bias may have stemmed from the difficulty of taking into account changes in value over time or from the known possible rewards for each target. Therefore, it is necessary to consider whether a similar bias occurs in situations where there is high uncertainty about value.

In situations in which obtaining information and executing an action exist simultaneously, two control strategies can arise: go-after-you-know and go-before-you-know. The go-after-you-know refers to situations in which the action is carried out after the goal has been determined, and the go-before-you-know refers to situations in which the action is started before the goal is determined^18^. These control strategies were studied in separate experimental settings. In particular, motor planning and execution in a go-before-you-know situation have been examined in which the final target is informed after the movement onset (the possible targets are known before the movement onset). However, in some situations, it is possible to either start a movement after determining the final target or to determine the final target while moving and correcting the ongoing movement. Therefore, a new decision-making problem arises as to how to make a decision between the control strategy of starting a movement after determining the target (i.e., go-after-you-know) or before determining the target (i.e., go-before-you-know).

As shown above, various decision-making problems need to be solved to improve performance. First, an individual needs to decide on a strategy (e.g., a simple reaction or a choice reaction) regarding the speed-accuracy tradeoff depending on a given time constraint. It is also necessary to determine the strategy in the time sequence of target determination and movement onset (e.g., go-after-you-know control or go-before-you-know control). Furthermore, the execution strategies in each control phase, such as the input system (how to see), the processing system (how to judge), and the output system (how to move), are also involved. However, it is still unclear how such a wide variety of decisions are made and what their optimality is, given the freedom to choose strategies.

**Figure 1:**
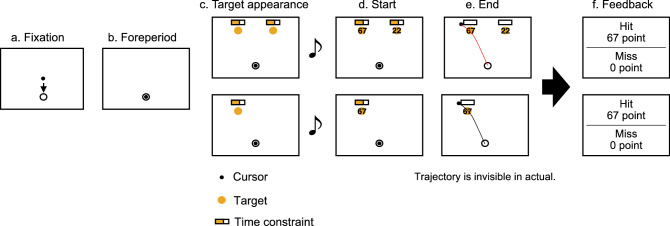
Trial sequences. The upper row shows the double-targets condition, and the lower row show the single-target condition. In all conditions, (**a**) at the beginning of a trial, the cursor was moved to the starting position. (**b**) After a 1-s delay period, (**c**) two targets or one target and an indicator showing time constraint were presented. After 1800–2200 ms of the target appearance, (**d**, **e**) the score was presented on each target with the auditory go signal, and the yellow range of the indicator starts to decrease. After the auditory go signal, the participant could perform the reaching movement freely. If the participants passed through the target within the time constraint, they received the score assigned to that target. (**f**) After the completion of the movement, participants were given feedback on their success, failure, and score.

The current study aims to clarify how sensorimotor strategies are selected under time constraints in the presence of uncertainty in the value of the motor targets and to examine the optimality of the selected strategy. The sequence of the experimental tasks is shown in Fig. 1. In our task, targets were assigned value, and participants could obtain that value when they reached it within a time constraint. In the main condition (the double-target condition), the values of the two targets were uncertain before the start of the trial, and the values of the targets were presented randomly for each trial in the range of 20–80 points with a beep to signal the start of the trial. The two targets were set at ± 15°, ± 22.5°, or ± 30° to the direction straight above the starting point and 20 cm away from the starting point. Participants were instructed to intend to maximize the mean score (total score/number of trials) in each set; to achieve this goal, the participants were allowed to freely decide on a strategy. Participants received a score for the target if they could reach the target within the time constraint assigned for each trial. To evaluate the optimality and the control strategy, we compared the behaviors in the double-target condition with those in the condition where only one target exists, which was performed as the control condition. From the interpretation of the behavioral patterns and the evaluation of the optimality of the strategies using a descriptive approach, we examined the mechanism of selecting a strategy for executing the behavior under time constraints and its optimality in a situation where there is uncertainty in value.

The possible actions in the double-target condition could be classified into simple reaction and choice reaction. In the case of “simple reaction,” the participant was expected to move toward the target in an almost straight trajectory with a short reaction time (RT). This strategy corresponds to the behavior when one target is completely ignored, which is consistent with the behavior in the single-target condition. In the case of “choice reaction,” a wide range of possible actions were predicted to be taken. For instance, the participant could choose one target over a longer RT than that in the simple reaction, then move toward that target in an almost straight line (go-after-you-know). Two other possible behaviors were considered as movement onset in incomplete target valuation. One is to initiate an intermediate direction between targets and then decide on a target and move towards it (go-before-you-know). The other is to initiate a movement toward one target and then make corrections as needed (go-after-you-know with motor target correction). Notably, the simple reaction shortens the arrival time without considering the target value, and the choice reaction increase the probability of reaching a higher value target even if the arrival time was longer. Therefore, the difference between these strategies would clearly appear in the two behavioral indices of reach timing and probability of reaching a high-value target. We could also expect that the time constraint may modulate the selection between these strategies; furthermore, the longer the time constraint, the greater the choice reaction. Our previous study^10^ has shown that in a two-choice response task under time constraints, there is a bias to use a choice reaction even in situations where a simple reaction is desirable. Thus, we confirmed whether the same bias was observed in the current task.

## Results

### Summary of methods and hypotheses

Participants (*N* = 12) performed single-target and double-target trials. The unique setting of the current task was that the target value was presented at the same time as the start signal of the task, and that a different time constraint was assigned in each trial. Thus, to obtain better outcomes, participants needed to decide whether to determine a better target and take action to target it or to reach one of the targets without determining the value of the target, depending on the given time constraints.

The single-target and double-target trials were performed in a random order for each trial. The reason for this random sequence was to eliminate the possibility that behavior in the conditions of previous and posterior trials was influential (a problem that exists when using a block design) when differences in movement patterns between the two conditions in response to time constraints were present. The current study mainly aimed to examine the effects of time constraints on decision-making and motor execution strategies. The single-target trials were set as a control condition to examine whether to utilize the benefits of having two targets effectively. The participants performed 324 trials (54 trials × 6 sets) of the task. Each set contained 36 trials in the double-target trials and 18 trials in the single-target trials. The directions of the targets were assigned evenly.

### Modulation of movement patterns depending on time constraints and target-separation-angles

**Figure 2:**
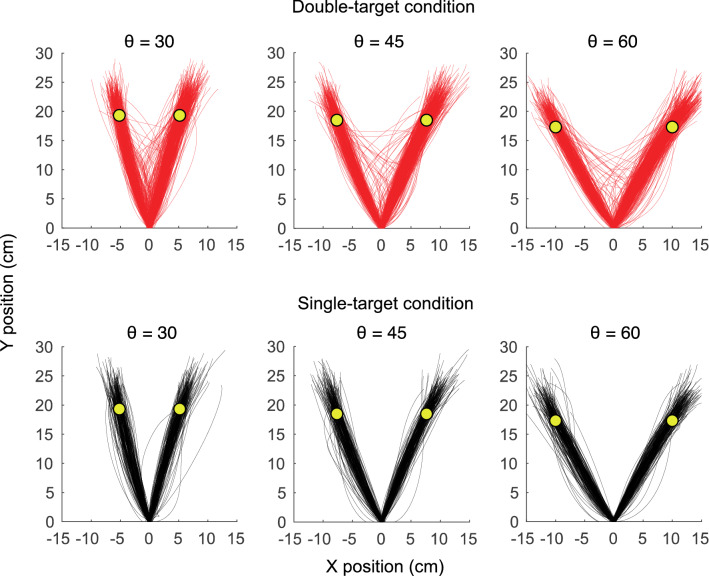
Trajectories in each target configuration. The cursor trajectories including all participants and all trials for each target configuration are shown. The upper row shows the double-target condition, and the lower row shows the single-target condition. In the single-target condition, participants made a linear trajectory, which is widely observed in general reaching movements. In contrast, in the double-target condition, although participants made many linear trajectories, there are trials in which participants selected a movement direction closer to the middle and trials in which the target is modified after the movement onset.

The cursor trajectories for each condition are shown in Fig. 2. In the single-target condition, the trajectories were approximately straight, which is widely observed in a general reaching movement. In contrast, in the double-target condition, there were trials that started in the direction of movement closer to the center. This difference in trajectories indicates that the decision-making phase affects movement trajectories.

**Figure 3:**
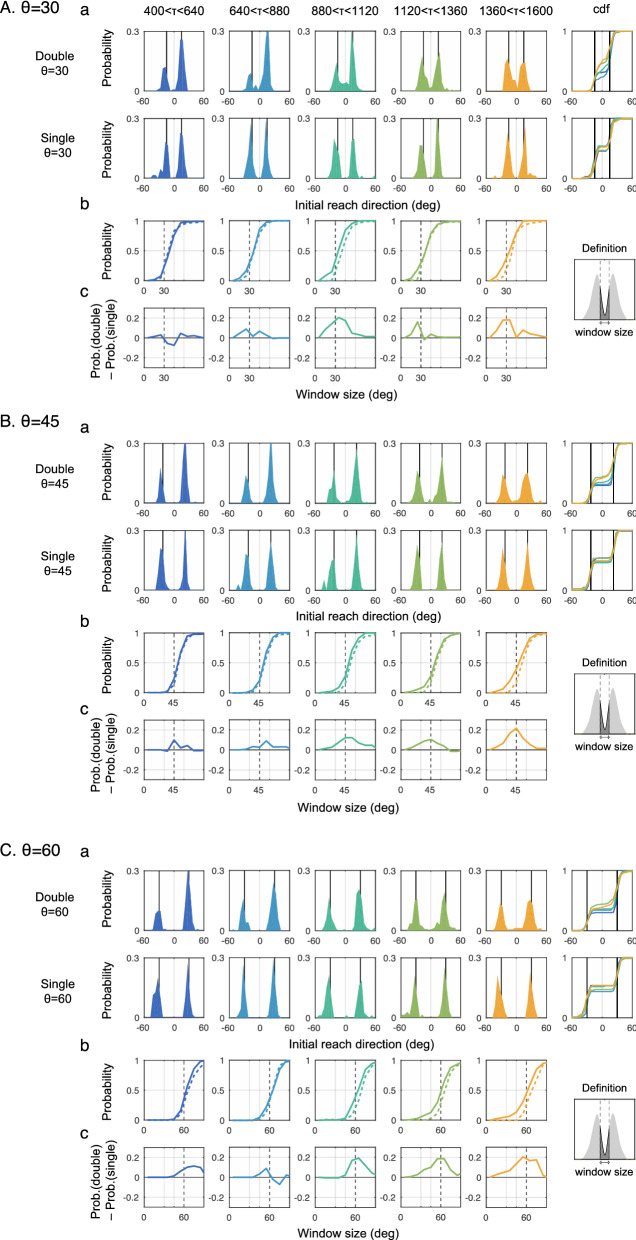
Initial movement direction according to time constraints. Panels (**A**), (**B**), and (**C**) show the conditions where the target-separation-angles were 30°, 45°, and 60°, respectively. (**a**) In each panel, the time constraints were classified into five bins, and the histograms and cumulative distribution functions (cdf) of the initial reach direction (i.e., the direction of the cursor after 100 ms) within each bin are shown in the two upper rows. The time constraints were classified into five bins, and the histograms and cumulative distribution functions (cdf) of the initial reach direction (i.e., the direction of the cursor after 100 ms) within each bin were shown. The color of the cumulative distribution function (rightmost column) corresponded to the color of the histogram for each time constraint. In the single-target condition, the histogram of the initial reach direction did not show any considerable change in response to the time constraint. This was manifest in the cdf, which was essentially flat in the range of movement directions between the two targets. In contrast, in the double-target condition, the distribution kurtosis corresponding to each target direction decreased and the frequency of initial movements directed toward the center increased in the long time-constraint condition compared to the short time-constraint condition. Similarly, from the cdf, it was confirmed that the frequency of the initial movement directed between targets varied with the time constraint, and the cdf had a positive slope in the range of movement directions between the two targets for the longer time constraints. (**b**) These panels showed the cumulative probability included in a window centered at 0 (the central direction) as a function of window size set to range from 0 to 90. The solid/dashed lines represent the double-target/single-target condition, respectively. If the solid lines were above the dashed lines, the frequency of the initial movements contained in the window was high. (**c**) The differences of cumulative probability functions (shown in **b**) between the double- and single-target conditions are shown. If the value was positive, the probability included in the window was high for the double-target condition than for the single-target condition.

**Figure 4:**
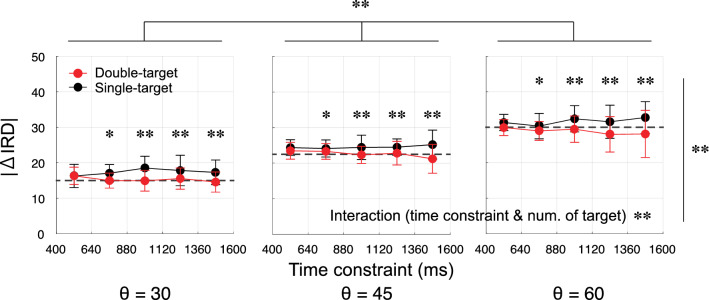
Comparison of the absolute value of ΔIRD between the double-targets condition and the single-target condition. Between-participant means and standard deviations of |ΔIRD| according to time constraint were shown for each target-angle condition. The ΔIRD denotes the deviation from the middle direction of the initial movement direction. The dashed line on each panel represents the |ΔIRD| for a direct reaching toward the target (e.g., at 15° for the 30° separation targets). Notably, |ΔIRD| below the dashed line indicates a more centrally directed movement. Two-way repeated-measures ANOVAs (time constraint [5] × number of targets [2]) on the |ΔIRD| in each target-angle condition showed a significant main effect of target condition in all angle conditions. The results of the multiple comparison tests showed that there was a significant difference when the time constraint was long. These results suggested that initial movements were more centering in the long time constraint condition. *: *P* < 0.05, **: *P* < 0.01.

The time constraints were classified into five bins, and the histograms of the initial reach direction (IRD) within each bin are shown in Fig. 3. The IRD indicates the direction from the start position to the cursor 100 ms after the onset of movement. This variable is often used to describe the characteristics of the initial movement in a go-before-you-know situation^19,20^. In the single-target condition, the histogram of the IRD did not change considerably with the time constraint, whereas in the double-target condition, the IRD was closer to the middle, and this tendency was observed as the time constraint became longer. Figure 4 shows a comparison between the double-target and single-target conditions in terms of |ΔIRD| (i.e., the magnitude of deviation of the IRD from the middle) according to the time constraint in each angle condition. A three-way repeated-measures ANOVA (time constraint [5] × number of targets [2] × target-separation angle [3], the statistical values were shown in Table 1 in the supplementary information) on the |ΔIRD| showed significant main effects of the number of targets (*P* < 0.001) and target-separation angle (*P* < 0.001). There was a significant interaction between time constraint and number of targets (*P* = 0.006) Post-hoc test showed a simple main effect of number of targets in the four longer time constraints. These results indicated that initial movements in the double-target condition were more centered than those in the single-target condition.

**Figure 5:**
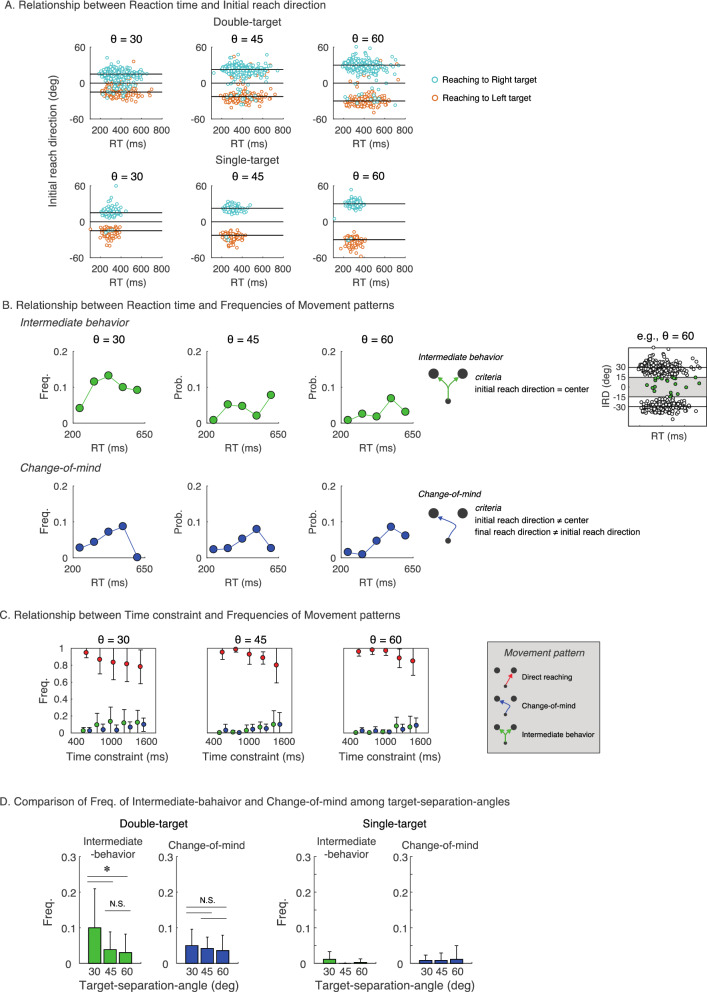
Modification of movement patterns corresponding to reaction time and time constraints. (**A**) These figures showed the relationship between reaction time and initial reach direction. The cyan circles represent the trials that reached the right target, and the orange circles represent the trials that reached the left target. The upper row represents the double-target condition, and the lower row represents the single-target condition. From these figures, the intermediate behaviors and movements with longer reaction times were confirmed in the double-target condition compared to the single-target condition. (**B**) The upper three panels from the left show the relationship between RT and frequency of intermediate behavior at each target-separation angle. The lower three panels from the left show the relationship between RT and frequency of change-of-mind at each target-separation angle. As shown in the rightmost figure, the intermediate behavior was defined as movement in a direction within half of the target-separation-angle. The frequencies were calculated for each of the five bins of RT ranging from 200 to 650 ms, in which data from all participants were pooled. (**C**) The three panels from the left show the relationship between time constraints and frequencies of each movement pattern. As shown in the rightmost figure, based on the direction of the initial movement and the final arrival position, the participants’ behaviors were classified into three movement patterns. It was confirmed that the longer the time constraint, the lower the frequency of direct reaching and the higher the frequency of intermediate behavior and change-of-mind. (**D**) These figures show between-participants mean frequencies of intermediate behavior (green bars) and of change-of-mind (blue bars) in the double-target and single-target conditions. These figures show that, in the double-target condition, the frequency of intermediate behavior was larger for smaller target-separation-angle, whereas the frequency of change-of-mind was independent of target-separation-angle. In the single-target condition, these behaviors were rarely taken; therefore, this difference was not simply due to increased sensitivity to intermediate movements with the narrower angle.

Subsequently, whether the intermediate behavior frequency increases as the time constraint increased was verified, using the incidence of initial movement to the intermediate direction (i.e., intermediate behavior). Figure 5A shows the relationship between RT and IRD. In contrast to the single-target condition (shown in the bottom row in Fig. 5A), the double-target condition (shown in the upper row in Fig. 5A) showed that the initial movements' frequency toward between targets was higher. It could also be observed that the initial movements toward between targets were more likely to existent at RTs above a certain level. An intermediate behavior was defined as movements when the absolute value of IRD was less than one-fourth of the target-separation angle. To verify that longer the RT, the more intermediate behaviors were implemented, the probability of the occurrence of intermediate behaviors was described as a function of the RT [upper panels (Fig. 5B)]. Notably, in this analysis, all trials for all participants were pooled, and incidence rates were calculated in five separate bins ranging from 200 to 650 ms. These figures showed that the intermediate behaviors were observed more frequently with longer RT for all target-angle conditions.

The frequency of change-of-mind behaviors was also examined [lower panels (Fig. 5B)]. Change-of-mind denoted that the participants changed their aiming target during movement, and trials in which the initial movement direction and the final arrival target did not match were defined as trials in which change-of-mind occurred. The results showed that the frequency of change-of-mind tended to be highest at moderate RT. However, in the case of long RT, the need for change-of-mind might have disappeared since sufficient essential information for value judgments was collected before the movement onset.

In a similar perspective, the modulation in movement strategies in response to time constraints was examined. The participants’ behaviors were classified into three movement patterns: the direct reaching, the intermediate behavior, and the change-of-mind behavior (Fig. 5C). The frequency of each movement pattern was calculated as a function of the time constraint. Two-way repeated-measures ANOVA (time constraint [5] × target-separation angles [3]) on the frequency of direct reaching revealed significant main effects for both factors (time constraint: *P* = 0.009, target-separation angles: *P* = 0.024) and no significant interaction (*P* = 0.19). Post-hoc comparisons on target-separation angles revealed significant differences in 30$^\circ$ vs. 45$^\circ$ and 30$^\circ$ vs. 60$^\circ$ and no significant difference in 45$^\circ$ vs. 60$^\circ$. Post-hoc comparisons on time constraints revealed significant differences in 400 < τ < 640 vs. 1120 < τ < 1360 and 400 < τ < 640 vs. 1360 < τ < 1600, 640 < τ < 880 vs. 1360 < τ < 1600, and 880 < τ < 1120 vs. 1360 < τ < 1600, and there was no significant difference in other combinations.

Two-way repeated-measures ANOVA (time constraint [5] × target-separation angles [3], statistical values are shown in Table 2 in the supplementary information) on the frequency of intermediate behavior revealed significant main effects for both factors (time constraint: *P* = 0.011, target-separation angles: *P* = 0.027) and no significant interaction (*P* = 0.33). Post-hoc comparisons on target-separation angles revealed significant differences in 30$^\circ$ vs. 45$^\circ$ and 30$^\circ$ vs. 60$^\circ$ and no significant difference in 45$^\circ$ vs. 60$^\circ$. Post-hoc comparisons on time constraints revealed significant differences in 400 < τ < 640 vs. 1120 < τ < 1360 and 400 < τ < 640 vs. 1360 < τ < 1600, and no significant difference in other combinations.

Two-way repeated-measures ANOVA (time constraint [5] × target-separation angles [3], statistical values are shown in Table 2 in the supplementary information) on the frequency of change-of-mind revealed significant main effects for time constraint (*P* = 0.002), but neither the main effect for target-separation angle (*P* = 0.455) nor interaction (*P* = 0.748) reached significance. Post-hoc comparisons on target-separation angles revealed significant differences in 30$^\circ$ vs. 45$^\circ$ and 30$^\circ$ vs. 60$^\circ$ and no significant difference in 45$^\circ$ vs. 60$^\circ$. Post-hoc comparisons on time constraints revealed significant differences in 400 < τ < 640 vs. 1120 < τ < 1360 and 400 < τ < 640 vs. 1360 < τ < 1600, and there was no significant difference in other combinations.

Figure 5D showed the frequency of each movement pattern depending on target-separation-angles. To examine whether the frequency of movement patterns changes with target-separation-angle, all time contractions were pooled, and the mean frequency of each pattern was calculated for each participant in each target-separation-angle. One-way repeated-measures ANOVA (statistical values are shown in Table 3 in the supplementary information) revealed that there was a significant main effect of target-separation-angle on intermediate behavior (*P* = 0.023), but there was no significant effect on change-of-mind (*P* = 0.464). Post-hoc comparison revealed significant differences in 30$^\circ$ vs. 45$^\circ$ and 30$^\circ$ vs. 60$^\circ$ and no significant difference in 45$^\circ$ vs. 60$^\circ$. These results suggested that the narrower the target-separation-angles were, the more intermediate behavior was taken. Notably, in the single-target condition, these behaviors were rarely taken; therefore, this effect of target-separation-angles on intermediate behavior was not due to increased sensitivity to intermediate movements with the narrower angle.

These results showed that the frequency of intermediate behavior and change-of-mind increased with longer time constraints. Correspondingly, the frequency of direct reaching decreased with longer time constraints. In addition, the frequency of intermediate behavior increased with a narrower target-separation-angle, and the frequency of direct reaching decreased accordingly, but change-of-mind was not affected by target-separation-angle.

### Comparison of spatio-temporal characteristics of behavior and performance between the double- and single-target condition

**Figure 6:**
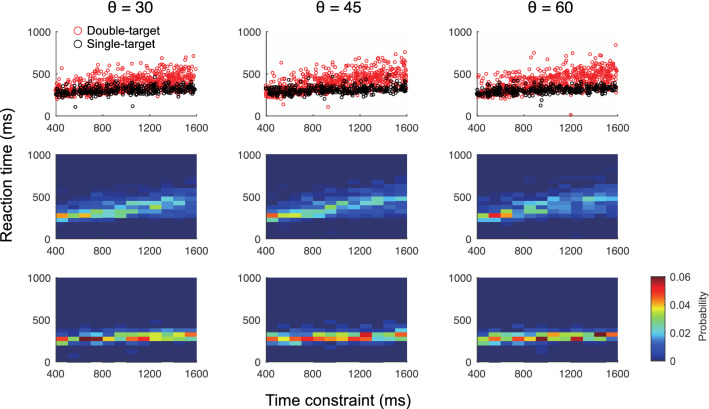
Comparison of reaction time according to time constraint between the double-target condition and the single-target condition. The upper panel shows a scatter plot of reaction times (of all participants) at a given time constraint. The middle and bottom rows show bivariate histograms (color maps) of time constraints and reaction times for the double-target and single-target conditions. These figures confirmed that the reaction times were comparable among the conditions when the time constraint was short, while the discrepancy in reaction times between the conditions increased as the time constraint became longer. Although the reaction times were similar between conditions under short time-constraints, the deviation of reaction times becomes larger as the time-constraint became longer.

To examine the changes in the sensorimotor strategy according to the number of targets and time constraints, the reaction time (RT) was compared among the conditions (Fig. 6). The upper panel of Fig. 6 shows a scatter plot of time constraints and RT, and the middle and lower panels show bivariate histograms of time constraints and RT in the double-target and single-target conditions. From these results, it can be confirmed that the RT was comparable between the double- and single-target conditions when the time constraint is short, while the deviation of the RT between the conditions increased as the time constraint becomes longer.

**Figure 7:**
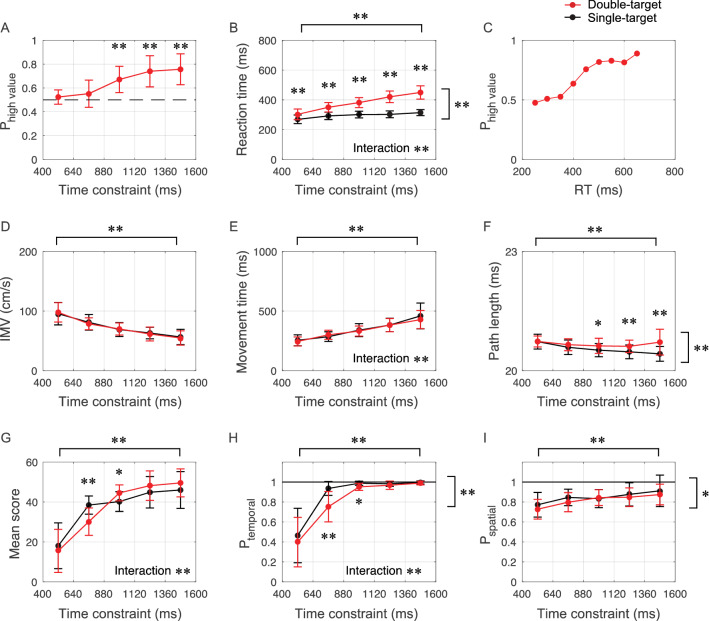
Comparison of execution strategy and performance according to time constraint between the double-target condition and the single-target condition. (**A**, **B**, **C**) The top row showed the probability of reaching a target with a higher score according to the time constraint (P_high-value_), the reaction time according to the time constraint (RT), and the P_high-value_ according to the reaction time. These panels show that the longer the time constraint, the larger the difference between the RT in the double-target and single-target conditions, and that longer RT in the double-target condition increased the probability of reaching a higher-value target. (**D**, **E**, **F**) The middle row showed the initial movement velocity (IMV), the movement time (MT), and the path length of the trajectory at a 20-cm Euclidian distance from the start position (PL) according to the time constraint. These panels indicate that the differences between the double- and single-target conditions did not significantly affect the time or speed of the movement. In addition, the trajectories were longer in the double-target condition than in the single-target condition when the time constraint was long. (**G**, **H**, **I**) The bottom panel showed the mean score, the temporal accuracy (P_temporal_), and the spatial accuracy (P_spatial_) according to the time constraint. The mean scores were higher in the double-target condition than in the single-target condition under a moderately tight time constraint, suggesting that the participants were not able to switch appropriately between simple and choice reactions. This was also supported by the fact that the P_temporal_ was lower in the double-target condition than in the single-target condition under moderately tight time constraints. *: *P* < 0.05, **: *P* < 0.01.

The top row in Fig. 7 shows the probability of reaching the target with the higher score (P_high-value_) according to the time constraint, RT according to the time constraint, and P_high-value_ according to the RT. The middle row shows the comparison of the initial movement velocity (IMV), movement time (MT), and path length (PL) of the trajectory at a 20-cm Euclidian distance from the start position (PL) according to the time constraint. The PL is a type of indicator of the curvature of movement. If the PL was exactly 20 cm, the movement was perfectly straight, and if it was higher than that, it indicated that the movement was performed in an indirect way. The bottom row shows the comparison among conditions of the mean score (mean score), temporal accuracy (P_temporal_), and spatial accuracy (P_spatial_) according to the time constraint. Notably, temporal success or failure was judged based on whether the arrival time was shorter than the provided time constraint, and P_temporal_ denotes temporal success probability. Spatial success or failure was judged based on whether the shortest distance between the target and cursor was smaller than the target radius (1 cm), and P_spatial_ denotes spatial success probability. These figures were constructed by including all target angle conditions.

P_high-value_ was defined as the probability of going to a higher value target, and if this was higher than the chance level (0.5), it could be interpreted that the participant performed in a choice reaction. One-sample t-tests (statistical values are shown in Table 4 in the supplementary information), with the P_high-value_ (Fig. 7A) as the dependent variable, confirmed significant differences above chance levels (0.5) in the latter three time-constraint levels (*P-values* < 0.001), suggesting that a choice reaction strategy is used in conditions with long time constraints. The RT was also a factor that explained the difference between simple and choice reactions. Two-way repeated-measures ANOVA (time constraint [5] × number of targets [2]) on the RT (Fig. 7B) revealed significant main effects for both factors (number of targets: *P* < 0.001; time constraint: *P* < 0.001). Importantly, a significant interaction (*P* < 0.001) was also identified, indicating that the difference in strategy between single-target and double-target varied with the time constraint. Post-hoc tests revealed the simple main effects of the number of targets (*P-values* < 0.001) in all time-constraint levels. The RT and P_high-value_ (Fig. 7C) had an relationship similar to that of a sigmoid curve, with P_high-value_ approaching the chance level when the RT was short and P_high-value_ approaching 1 as the RT increased. Since a sigmoidal relationship between the RT and P_high-value_ (Fig. 7C) and a linear relationship between RT (Fig. 7B) and time constraint were observed, the P_high-value_ had an increasing pattern similar to a sigmoidal curve with the time constraint (Fig. 7A).

The results for IMV and MT as indicators of characteristics with respect to the speed of movement are shown in Fig. 7D, E. A two-way repeated-measures ANOVA (time constraint [5] × number of targets [2], statistical values are shown in Table 5 in the supplementary information) on the IMV (Fig. 7D) showed a significant main effect for time constraint (*P* < 0.001), no significant main effect for number of targets (*P* = 0.74), and no significant interaction (*P* = 0.29). A two-way repeated-measures ANOVA (time constraint [5] × number of targets [2]) on the MT (Fig. 7E) showed a significant main effect for time constraint (*P* = 0.047), no main effect of the number of targets (*P* = 0.43), and a significant interaction (*P* = 0.047). Post-hoc tests revealed simple main effects of time constraints in both the double-targets condition and single-target condition (*P-values* < 0.001) and no simple main effects of the number of targets conditions at all time constraint levels. These results indicate that the number of alternatives did not significantly affect IMV and MT, although these values changed with time constraints. In other words, there was no change in movement speed that would compensate for the change in RT due to the difference in the number of targets.

The result for PLs as indicators of characteristics for the deviation from the direct path is shown in Fig. 7F. If the participant made a completely direct path, this value would be 20 cm (equal to Euclidean distance from the start position to target), but if a detour was taken, the value would be greater than 20. Two-way repeated-measures ANOVA (time constraint [5] × number of targets [2], statistical values are shown in Table 5 in the supplementary information) on the PL (Fig. 7F) showed a significant main effect for both time constraints (*P* < 0.001) and number of targets (*P* = 0.005). Importantly, a significant interaction (*P* < 0.001) was also found. Post-hoc tests revealed simple main effects of time constraint in the single-target condition (*P* < 0.001). There were simple main effects of the number of targets conditions only in the longer three time-constraint ranges (880 < τ < 1120: *P* = 0.042; 1120 < τ < 1360: *P* = 0.001; 1360 < τ < 1600: *P* = 0.005). These results suggest that different movement patterns were used between the double-target and single-target conditions as the time constraint increased. It should also be emphasized that the range of time constraints in which this difference occurred coincides with |ΔIRD| (Fig. 4) and the increase in the frequency of change-of-mind and centering initial movement (Fig. 5C).

Two-way repeated-measures ANOVA (time constraint [5] × number of targets [2], statistical values are shown in Table 5 in the supplementary information) on the mean score (Fig. 7G) showed a significant main effect for time constraint (*P* < 0.001) and no significant main effect for the number of targets (*P* = 0.952). A significant interaction (*P* < 0.001) was found. Additionally, there were simple main effects of time constraint in both types of target conditions (*P-values* < 0.001). There were simple main effects of the number of targets conditions only in the second and third time-constraint ranges (640 < τ < 880: *P* = 0.002; 880 < τ < 1120: *P* = 0.016). Two-way repeated-measures ANOVA (time constraint [5] × number of targets [2], statistical values are shown in Table 5 in the supplementary information) on the P_temporal_ (Fig. 7H) showed significant main effects for both factors (number of targets: *P* = 0.002; time constraint: *P* < 0.001). A significant interaction (*P* < 0.01) was also found. There were simple main effects of time constraints in both types of target conditions (*P-values* < 0.001). There were simple main effects of the number of targets conditions only in the second and third time-constraint ranges (640 < τ < 880: *P* = 0.001; 880 < τ < 1120: *P* = 0.017). Two-way repeated-measures ANOVAs (time constraint [5] × number of targets [2], statistical values are shown in Table 5 in the supplementary information) on the P_spatial_ (Fig. 7I) showed significant main effects for both factors (number of targets: *P* = 0.027; time constraint: *P* < 0.001). There was no significant interaction (*P* = 0.74). These results suggest that, compared to situations in which the goal is predetermined, situations in which choices exist have a negative effect on temporal (especially in moderately tight time constraints) and spatial (in a wide range of time constraints) performance.

## Discussion

The current study examined a sensorimotor strategy under time constraints in a situation where there was uncertainty about the value of the target. In the main task, there were two alternative targets, and target values were presented along with an auditory go signal that indicated the start of the trial. The target value of each target was not shown until after the go signal and participants had to reach the target within a time constraint that could vary from trial to trial. When the time constraint is short, it is desirable to adopt a simple-reaction strategy because the attempt to obtain information about the target’s values will not result in reaching the target in time. In contrast, if there is sufficient time to process the target values, it is preferable to accurately recognize the target information and choose the target that has a higher value (i.e., a choice-reaction strategy). Thus, time constraint is a key factor that determines the desired strategies for the participants. Moreover, in the current task, the participants were free to initiate actions at any time after the auditory go-signal, so they could start movements after identifying the target values or before identifying them. This setup allows us to evaluate the optimality of the strategy and examine the characteristics of the selected movement patterns.

By analyzing the modulations in spatiotemporal movement patterns according to time constraints, we obtained three main findings. First, the modulation of the initial reach direction and the PL of trajectories under the time constraint revealed that different motor patterns were selected between the double-target and single-target conditions when the time constraint became relatively long. Second, the motor trajectories were modulated depending on both the time constraint and the interval between targets. Specifically, the longer the time constraint, higher the frequency of behaviors, such as intermediate behavior and change-of-mind, due to the movement onset in an incomplete state of target selection. Third, participants used the choice-reaction strategy even in the tightly time-constrained condition, and their performance was lower than that in the condition with a single goal. Since the temporal performance (i.e., whether it reached in time) was significantly lower, this reduced performance in the double-target condition was caused by selecting the choice reaction despite not being able to meet the time constraint. We could not compare the participants’ behavior with the complete optimal solution, but the lower performance in the double-target condition compared to the single condition indicated that the participants’ behavior was at least suboptimal. Moreover, we should emphasize the tendency to use the choice reaction even in situations where the simple reaction was clearly better and not vice versa. This suggested that a bias existed in the participants’ strategy selection.

### Modulation of movement pattern depending on time constraints and target-separation-angle in the double-target condition

It is generally known that in a reaching movement, the trajectory is almost straight to the target^21,22^. In fact, a trajectory of approximately straight movement was often observed in the single-target condition, and if the movement was initiated after the determination of the target, it was likely that the movement would also follow a straight trajectory in the double-target condition. However, we found that the initial reach direction was closer to the middle direction of the two targets under a relatively long time constraint, suggesting that the valuation process between the targets may have interfered with the movement trajectory (Figs. 3 and 4). The analysis for movement patterns found that the frequency of intermediate behavior and change-of-mind increased with increasing time constraints, whereas the frequency of direct reaching decreased (Fig. 5). In addition, although intermediate behavior was more likely to occur when the target-separation angle was larger, change-of-mind was not affected by the target-separation angle. The narrower the target-separation angle, the more intermediate initial movement occurred, which was comparable to results of a previous study^23^. These results suggest that both time constraints and target location affected the participants’ strategies.

The question that arises here is whether this interference is due to unintentional or intentional control, but a view that supports each is possible. Many previous studies have reported that when initiating a movement in the presence of multiple potential targets, the initial movements are directed toward the weighted average of the given targets^19,23–28^. An unintentional averaging output of discrete motor plans corresponding to each potential target has been proposed as one of the causes of such motor patterns. This is supported by neurological evidence that motor plans for each target are represented simultaneously when there are multiple competing movement targets in the reaching-related areas^29–33^.

Most of the studies that provided this evidence used go-before-you-know situations, in which participants were forced to initiate a movement for multiple competing targets and then reach a final goal presented after the movement had begun^19,23,24,26,27,34–38^. However, the present study was different from the go-before-you-know situation in that the participants were able to initiate the movement at their own timing. Therefore, it was also possible to start a movement after the movement target had been specified by the participants. Even in such a situation, we observed the centering of the initial movement in part of trials, which may be attributed to the existence of some advantages for online control to achieve a better goal during a movement^39–45^, as detailed below.

One of the possible reasons for the intermediate initial movement is that the participants may have intended to shorten the reaction time since the participants were required to reach the target within a given time constraint and thus, there is an advantage of starting earlier. As shown in a previous study, an overlap between motor planning and execution reduces the reaction time when there are multiple motor goals^40^. In addition, reaction time and movement time can be reduced if participants pre-plan the kinematic components of the movements commonly required for both targets before determining the targets^46^. Thus, it is possible that the centralized initial movement is a goal-oriented action generation that increases the time available to determine the movement target^42^.

Another possibility of the centering tendency of the initial movement is to maintain the capability to respond to target changes during movement execution. Accounting for possible movement corrections that may occur later is an important aspect of motor planning^43^, and the state of the movement affects whether or not the goal is changed^44,45^. Because the current task requires instantaneous decisions, errors in value processing may occur, and changing the target during movement execution may be necessary. It was reported that the intermediate behavior minimizes the motor costs associated with corrections required after recognizing the final target^47,48^, suggesting that intermediate behavior is advantageous when dealing with deciding or changing target during movement. In a previous study, it was reported that the benefit of the intermediate behavior decreased as the target-separation angle became wider, leading to a decrease in the frequency of the intermediate behavior^23^. The current study showed the same tendency, suggesting that the intermediate behavior was used more actively in situations where its advantages were significant. The intermediate behavior could reduce the cost of possible later movement corrections, including temporal performance, reach accuracy, and biomechanical costs.

Meanwhile, there was another adaptive behavior: change-of-mind. Similar to the intermediate behavior, the frequency of change-of-mind was also shown to be more likely to occur with longer time constraints. Previous studies have reported that even after the initial decision to initiate a movement, valuation processing is conducted to reverse or reaffirm the initial decision^49–52^. The behavior in the current study can be viewed similarly. It was considered that participants perform value judgments and reassessments during movement if they have sufficient time; conversely, if they did not have sufficient time, reassessments after deciding on one target or the other are suppressed. The relationship between RT and change-of-mind frequency showed an interesting trend, with the frequency of change-of-mind being highest at moderate RT. This modulation suggests that there was a motor pattern phase with cognitive and motor overlap between simple and complete choice reactions (i.e., correct go-after-you-know). In other words, it was considered that in moderate RT trials, the value evaluation and goal determination before the movement initiation was incomplete, and the goal may need to be revised due to overlapping value representations even during the movement.

Although previous studies have reported that the incidence of change-of-mind is affected by the movement cost^53^, the change-of-mind frequency was not affected by the angle in the current study. This result may be due to the following: the effect of the target-separation angle on the success rate and cost of change-of-mind was small in the range of this study, or the frequency (situation in which change-of-mind is necessary) was small in the first place. Thus, the effect of the target-separation angle could not be detected. In addition, previous studies have shown that the dynamic state during movement (e.g., velocity or acceleration) and the difference of values between targets explain the frequency of change-of-mind^44^. Such behavior might exist in the present study, but it was difficult to analyze the relationship between the motor state and the occurrence of change-of-mind in detail because the number of trials in which change-of-motion occurred was small. In a future study, it would be worthwhile to examine whether participants control their movements considering the change-of-mind possibility subsequently (i.e., whether change-of-mind is performed reactively or whether the change-of-mind possibility was taken into account in the movement planning). For example, in the current task, the change-of-mind possibility was different between movement initiation toward a target with a score of 75 points and toward 50 points. Therefore, the difference in the possibility of change-of-mind estimated by the value of the initial aiming target may affect initial movement kinematics and gaze behavior to search target values during movement. Overall, in the current study, it was shown that the frequency of change-of-mind modulates with RT and time constraints, which may be the result of adaptive behavior reflecting the conflict between temporal demands and value selection to reach a better option.

### Suboptimal strategy selection and bias to prefer choice reaction in the double-target condition

When the time constraint became longer, the reaction time was longer in the double-target condition than in the single-target condition, while the reaction times were close to an intermediate value between the conditions under severe time constraints. Accompanying this change, the probability of reaching the target with the highest score (P_high-value_) increased as the time constraint increased. Therefore, when the reaction time was short, the participant started to move in the predetermined direction as a simple reaction, but when the reaction time was long, the participant started to move after acquiring the value information as a choice reaction. These results showed that the participants varied their strategy to obtain information about the target depending on the given time constraint.

The question is whether such a strategy switch is optimal or biased in terms of objective reward maximization. When we compared the mean scores according to the level of time constraint between the single-target and double-target conditions, interestingly, we found that participants performing the single-target condition scored higher under the relatively severe time constraint. This tendency was consistent across participants and can be attributed to two factors. The first is the difference in temporal performance. In the double-target condition, the probability of reaching the target in time was lower than that in the single-target condition under the relatively more severe time constraint. The lower temporal performance reflects, in the double-target condition, the longer reaction time due to the time spent obtaining information about the target and the lack of a corresponding reduction in the movement time. Second, the difference in the expected value between simple and choice reactions is not significantly large in theory. In a simple reaction, since each target is a random value between 20 and 80, the expected value of the target that the participant aims at is 50 points, even if a movement completely succeeds in temporal and spatial. On the other hand, if the participant performs a choice reaction and selects and heads for the higher scoring of the two targets with perfect accuracy, the expected value of the target is 60 points. Since the difference in the expected value between reaction patterns is theoretically less than 10 points, if the success probability of the double-target condition is less than 5/6 against the success probability of the single-target condition, rather than benefiting from making a choice, a loss is incurred.

Interestingly, the preference of the choice-reaction strategy over the simple-reaction strategy, even when the benefit of the strategy is small, is a cognitive bias similar to the tendency of our previous study to have longer reaction times than the optimal strategy^10^. In the present study, avoiding the uncertainty of the outcome possibly caused the preference for the choice-reaction strategy. In the presence of two different values, it is undesirable to start a movement with uncertainty about which value target to go for, and this may lead to a cognitive bias in choosing the better alternative.

Many previous studies have found that risk-seeking decision-making is more likely to occur in motor tasks^6,7,54–56^, which can be interpreted from two perspectives: distortion of subjective and objective values (i.e., preference for higher scores) and overestimation of one’s own ability. The distortion of subjective and objective values has been widely confirmed in economic decision-making^57^ and suggested to be present in motor tasks as well, although it was shown to be approximated by different functions^5^. There is a tendency to choose the highest value that exists in the place. In such cases, it is acceptable to use a choice strategy by selecting the option with the higher reward. In particular, motor tasks may be prone to such preferences, because daily movement decisions require instantaneous decisions.

Another possibility, as mentioned above, is the existence of a bias in the estimation of ability. In the current task, it is necessary to correctly estimate the relationship between reaction time and choice accuracy, but it is not clear whether humans are able to represent the relationship accurately. In the current task, it is necessary to correctly estimate the relationship between reaction time and accuracy of choice, as well as the relationship between the movement time and the reach accuracy; however, it is not clear whether humans can represent them correctly. Given the representation that it is possible to make accurate choices even with a shorter reaction time than actual, it is understandable that the choice-reaction strategy is used frequently in a suboptimal manner.

Presently, the major challenge is the difficulty in separating the effects of distortion of subjective and objective values and misestimation of one's own abilities. It is possible that only one influence is strongly at work, or that both are at work, or that different factors are at work depending on the decision-maker. In our previous study^10^, we found that different strategies were selected between conditions in which the scores changed and conditions in which the probabilities changed, even when the expected values were equal. Thus, in future studies, manipulating value from various factors and examining the modulation of the strategy accordingly may contribute to the separation of the two possibilities.

### Conclusion

The current study investigated a sensorimotor strategy according to a time constraint under uncertainty about the target values. We obtained three main findings. First, the modulation of the movement kinematic patterns under time constraints revealed that different motor patterns were selected between the double-target condition and single-target conditions when the time constraint became relatively long. Second, the motor patterns were modulated by the time constraint and the interval between targets. The longer the time constraint, the higher the frequency of behaviors, such as intermediate behavior and change-of-mind, due to the movement initiation in an incomplete target selection state. This modulation could be due to both unintentional and intentional control. Even then, more importantly, it can be interpreted as an adaptive action reflecting the conflict between temporal demands and value selection to reach a better option. Third, we found that performance was consistently lower than that in the single-goal condition across participants since participants frequently used the choice-reaction strategy even in the tightly time-constrained. The results suggest that there is a consistent cognitive bias among individuals to choose a higher value in situations where there are multiple alternatives with different values. Future studies are required to clarify the causes of this bias.

## Methods

### Participants

Twelve right-handed participants (age: 21.3 $\pm$ 2.3 years, ten male) were recruited. All participants had normal or corrected-to-normal vision. All participants were naive to the purposes of this study and provided written informed consent. This study was approved by the Ethics Committee of the Graduate School of Arts and Sciences, University of Tokyo. All experimental procedures adhered to approved guidelines. Written informed consent was obtained from each participant.

### Experimental setup

The participants sat in a quiet, dim room. A pen tablet with sufficient workspace to measure the participant’s arm reach movement (Wacom, Intuos 4 Extra Large; workspace: 488 × 305 mm) was set on the table. A monitor (I-O DATA, KH2500V-ZX2, 24.5 inches, 1920 × 1080 pixels, vertical refresh rate 240 Hz) that was used to present stimuli was set with an approximately 30º gradient angle over the pen-tablet. The participants manipulated a cursor on a screen whose position was transformed from the position of the pen. The elapsed time from the movement onset and the location of the cursor on the monitor were sampled at 240 Hz. All stimuli were controlled using the Psychophysics Toolbox of MATLAB (MathWorks, Natick, MA, USA).

### Experimental task

The participants performed the double-target trials as the main condition and the single-target trials as the control condition. The trial sequences for both conditions are shown in Fig. 1. In the double-target trials, the two targets (radius = 1 cm) were set at ± 15°, ± 22.5°, and ± 30° to the direction directly above the starting point (target separation angle θ = 30°, 45°, and 60°, respectively) and 20 cm away from the starting point. Before starting the trial, the time constraint (the bar on the target) and the location of the target were shown on the screen. The time constraint for each target was randomly assigned for each trial in the range of 400–1600 ms, and participants were able to recognize the time constraint by the size of the yellow area. The values of the two targets were uncertain until the start of the trial, and the values were presented at the start. The target scores were randomly assigned in the range of 20 to 80 points for each trial and were presented as numbers on the target after trial onset. The bar indicated the time remaining after onset, the yellow area gradually shrank and finally disappeared. If the participant was able to cross the target while the yellow area remained, they were scored, and feedback was obtained after the trial. In single-target trials, only one target on either side was presented. Participants were instructed to maximize the mean score in each set. The mean score within a set was presented after each set was completed. The basic sequence of the task was the same as that for the main condition.

The participants performed 324 trials (54 trials × 6 sets) of the task. Each set contained 36 trials in the double-target trials (3 angles × 12 trials) and 18 trials (3 angles × 6 trials) in the single-target trials. The directions of the targets were assigned evenly. Between sets, participants receive sufficient rest to avoid fatigue.

### Data analysis

The last five sets (double-target trials [180 trials], single-target trials [90 trials]) were included in the analysis to exclude data from the initial adaptation process to the task. The cursor position (horizontal position: Xc(t), vertical position: Yc(t)) at each time (t) was smoothed using a second-order low-pass Butterworth filter with a cutoff frequency of 14 Hz.

Reaction time (RT) was defined as the time at which the cursor moved 1 cm away from the start position (this was also the criteria for movement onset). The arrival time (AT) was defined as the time when the cursor was more than 20 cm away from the starting point. The difference between AT and RT was defined as the movement time (MT). The cursor direction at each time (t) was defined as the angle formed by the vector from the start position to the cursor and the horizontal vector. The initial reach direction (IRD) was defined as the cursor direction 100 ms after movement onset (this timing has been used in previous studies^23,37^ examining the characteristics of initiation behavior), with 0° directly above, positive values in the clockwise direction (i.e., right), and negative values in the counterclockwise direction (i.e., left). |ΔIRD| is defined as the unsigned angle between the intermediate direction and IRD. The initial movement velocity (IMV) was defined as the movement velocity of the cursor (IMV: cm/s) 100 ms after movement onset. Temporal success or failure was judged based on whether the arrival time was smaller than the given time constraint. Spatial success or failure was judged based on whether the shortest distance between the target and cursor was smaller than the target radius (1 cm). The temporal success probability is denoted as P_temporal_, and the spatial success probability is denoted as P_spatial_. In the double-targets condition, the P_high-value_ was defined as the probability of reaching the target with the higher score of the two alternatives. The time constraints (minimum: 400 ms, maximum: 1600 ms) were classified into five equal time intervals, and within each bin, the mean values of the above variables were calculated for each participant.

The behavior in each trial was classified into three motor patterns based on the initial reach direction and the final reaching direction: “direct reaching,” “intermediate behavior,” and “change-of-mind.” The initial movement was defined as directed toward the target when the absolute value of IRD was greater than one-fourth of the target-separation angle and initial movement in the intermediate direction when it was less than one-fourth. The final reach direction was coded as either left or right. The direct reaching was a movement pattern in which the initial and final reach direction coincided. The intermediate behavior was a behavioral pattern in which the initial movement direction was intermediate. The change-of-mind was a behavioral pattern in which the initial and final directions were different. The frequencies of these movement patterns were calculated for each target-separation angle as a function of time constraint (400–1600 ms separated by five equal bins) and RT (200–650 ms separated by five equal bins). The frequencies at each target-separation angle were also calculated to examine the effect on the movement pattern selection.

### Statistical analysis

A three-way repeated-measures ANOVA (time constraint [5] × number of targets [2] × target-separation angle [3]) on the |ΔIRD| was conducted.

For frequency comparison of movement patterns, two-way repeated-measures ANOVAs (time constraint [5] × target-separation-angles [3]) on the frequencies of movement patterns (direct reaching, intermediate behavior, and change-of-mind) were performed. One-way repeated-measures ANOVAs (target-separation-angles [3]) on the frequencies of movement patterns (direct reaching, intermediate behavior, and change-of-mind) were performed.

We also performed two-way repeated-measures ANOVAs (time constraint [5] × number of targets [2]) on the mean values of the initial movement parameters (RT, IRD, IMV) and performance variables (MT, P_high-value_, P_temoral_, P_spatial_, score), including all angle conditions. In each target-angle condition, two-way repeated-measures ANOVAs (time constraint [5] × number of targets [2]) on the mean values of |ΔIRD| were conducted. Multiple comparison tests using the Bonferroni method were conducted between the double-target condition and the single-target condition at each time constraint level. Partial *η*^2^ for ANOVA and Cohen’s *d* for post-hoc *t*-tests were used to report effect sizes. *P*-values and summary of statistical results are reported in the Results section, and detailed statistics are reported in the supplementary information.

## Supplementary Information

Supplementary Tables.

## References

[1] Trommershäuser J, Mattis J, Maloney LT, Landy MS (2006). Limits to human movement planning with delayed and unpredictable onset of needed information. Exp. Brain Res..

[2] Landy MS, Goutcher R, Trommershäuser J, Mamassian P (2007). Visual estimation under risk. J. Vis..

[3] Hudson TE, Maloney LT, Landy MS (2008). Optimal compensation for temporal uncertainty in movement planning. PLoS Comput. Biol..

[4] Wu S-W, Trommershäuser J, Maloney LT, Landy MS (2006). Limits to human movement planning in tasks with asymmetric gain landscapes. J. Vis..

[5] Wu SW, Delgado MR, Maloney LT (2009). Economic decision-making compared with an equivalent motor task. Proc. Natl. Acad. Sci. U. S. A..

[6] Ota K, Shinya M, Kudo K (2015). Motor planning under temporal uncertainty is suboptimal when the gain function is asymmetric. Front. Comput. Neurosci..

[7] Ota K, Shinya M, Kudo K (2016). Sub-optimality in motor planning is retained throughout 9 days practice of 2250 trials. Sci. Rep..

[8] Ota K, Tanae M, Ishii K, Takiyama K (2020). Optimizing motor decision-making through competition with opponents. Sci. Rep..

[9] Dean M, Wu SW, Maloney LT (2007). Trading off speed and accuracy in rapid, goal-directed movements. J. Vis..

[10] Onagawa R, Shinya M, Ota K, Kudo K (2019). Risk aversion in the adjustment of speed-accuracy tradeoff depending on time constraints. Sci. Rep..

[11] Farashahi S, Ting CC, Kao CH, Wu SW, Soltani A (2018). Dynamic combination of sensory and reward information under time pressure. PLoS Comput. Biol..

[12] Fitts PM (1954). The information capacity of the human motor system in controlling the amplitude of movement. J. Exp. Psychol..

[13] Harris CM, Wolpert DM (1998). Signal-dependent noise determines motor planning. Nature.

[14] Heitz RP (2014). The speed-accuracy tradeoff: History, physiology, methodology, and behavior. Front. Neurosci..

[15] Chittka L, Skorupski P, Raine NE (2009). Speed-accuracy tradeoffs in animal decision making. Trends Ecol. Evol..

[16] Hick WE (1952). On the rate of gain of information. Q. J. Exp. Psychol..

[17] Hyman R (1953). Stimulus information as a determinant of reaction time. J. Exp. Psychol..

[18] Gallivan JP, Chapman CS, Wolpert DM, Flanagan JR (2018). Decision-making in sensorimotor control. Nat. Rev. Neurosci..

[19] Stewart BM, Gallivan JP, Baugh LA, Flanagan JR (2014). Motor, not visual, encoding of potential reach targets. Curr. Biol..

[20] Gallivan JP, Stewart BM, Baugh LA, Wolpert DM, Flanagan JR (2017). Rapid automatic motor encoding of competing reach options. Cell Rep..

[21] Flash T (1987). The control of hand equilibrium trajectories in multi-joint arm movements. Biol. Cybern..

[22] Morasso P (1981). Spatial control of arm movements. Exp. Brain Res..

[23] Haith AM, Huberdeau DM, Krakauer JW (2015). Hedging your bets: Intermediate movements as optimal behavior in the context of an incomplete decision. PLoS Comput. Biol..

[24] Chapman CS (2010). Reaching for the unknown: Multiple target encoding and real-time decision-making in a rapid reach task. Cognition.

[25] Gallivan JP (2011). One to four, and nothing more. Psychol. Sci..

[26] Stewart BM, Baugh LA, Gallivan JP, Flanagan JR (2013). Simultaneous encoding of the direction and orientation of potential targets during reach planning: Evidence of multiple competing reach plans. J. Neurophysiol..

[27] Krüger M, Hermsdörfer J (2019). Target uncertainty during motor decision-making: The time course of movement variability reveals the effect of different sources of uncertainty on the control of reaching movements. Front. Psychol..

[28] Carroll TJ, Mcnamee D, Ingram JN, Wolpert DM (2019). Rapid visuomotor responses reflect value-based decisions. J. Neurosci..

[29] Cisek P, Kalaska JF (2005). Neural correlates of reaching decisions in dorsal premotor cortex: Specification of multiple direction choices and final selection of action. Neuron.

[30] Klaes C, Westendorff S, Chakrabarti S, Gail A (2011). Article choosing goals, not rules: Deciding among rule-based action plans. Neuron.

[31] Coallier É, Michelet T, Kalaska JF (2015). Dorsal premotor cortex: Neural correlates of reach target decisions based on a color-location matching rule and conflicting sensory evidence. J. Neurophysiol..

[32] Cui H, Andersen RA (2011). Different representations of potential and selected motor plans by distinct parietal areas. J. Neurosci..

[33] Dekleva BM, Ramkumar P, Wanda PA, Kording KP, Miller LE (2016). Uncertainty leads to persistent effects on reach representations in dorsal premotor cortex. Elife.

[34] Hudson TE, Maloney LT, Landy MS (2007). Movement planning with probabilistic target information. J. Neurophysiol..

[35] Gallivan JP, Barton KS, Chapman CS, Wolpert DM, Randall Flanagan J (2015). Action plan co-optimization reveals the parallel encoding of competing reach movements. Nat. Commun..

[36] Gallivan JP, Bowman NAR, Chapman CS, Wolpert DM, Flanagan JR (2016). The sequential encoding of competing action goals involves dynamic restructuring of motor plans in working memory. J. Neurophysiol..

[37] Wong AL, Haith AM (2017). Motor planning flexibly optimizes performance under uncertainty about task goals. Nat. Commun..

[38] Nashed JY, Diamond JS, Gallivan JP, Wolpert DM, Flanagan JR (2017). Grip force when reaching with target uncertainty provides evidence for motor optimization over averaging. Sci. Rep..

[39] Gomi H (2008). Implicit online corrections of reaching movements. Curr. Opin. Neurobiol..

[40] de Xivry JJO, Legrain V, Lefèvre P (2017). Overlap of movement planning and movement execution reduces reaction time. J. Neurophysiol..

[41] Gallivan JP, Barton KS, Chapman CS, Wolpert DM, Flanagan JR (2015). Action plan co-optimization reveals the parallel encoding of competing reach movements. Nat. Commun..

[42] Wong AL, Haith AM, Krakauer JW (2014). Motor planning. Neuroscientist.

[43] Gallivan JP, Logan L, Wolpert DM, Flanagan JR (2016). Parallel specification of competing sensorimotor control policies for alternative action options. Nat. Neurosci..

[44] Marti-Marca A, Deco G, Cos I (2020). Visual-reward driven changes of movement during action execution. Sci. Rep..

[45] Michalski J, Green AM, Cisek P (2020). Reaching decisions during ongoing movements. J. Neurophysiol..

[46] Gallivan JP, Barton KS, Chapman CS, Wolpert DM, Flanagan JR (2015). Action plan co-optimization reveals the parallel encoding of competing reach movements. Nat. Commun..

[47] Christopoulos V, Schrater PR (2015). Dynamic integration of value information into a common probability currency as a theory for flexible decision making. PLoS Comput. Biol..

[48] Christopoulos V, Bonaiuto J, Andersen RA (2015). A biologically plausible computational theory for value integration and action selection in decisions with competing alternatives. PLoS Comput. Biol..

[49] Resulaj A, Kiani R, Wolpert DM, Shadlen MN (2009). Changes of mind in decision-making. Nature.

[50] van den Berg R (2016). A common mechanism underlies changes of mind about decisions and confidence. Elife.

[51] Wolpert DM, Landy MS (2012). Motor control is decision-making. Curr. Opin. Neurobiol..

[52] Wispinski NJ, Gallivan JP, Chapman CS (2018). Models, movements, and minds: Bridging the gap between decision making and action. Ann. N. Y. Acad. Sci..

[53] Moher J, Song JH (2014). Perceptual decision processes flexibly adapt to avoid change of mind motor costs. J. Vis..

[54] Nagengast AJ, Braun DA, Wolpert DM (2011). Risk sensitivity in a motor task with speed-accuracy trade-off. J. Neurophysiol..

[55] O’Brien MK, Ahmed AA (2013). Does risk-sensitivity transfer across movements?. J. Neurophysiol..

[56] Mamassian P (2008). Overconfidence in an objective anticipatory motor task. Psychol. Sci..

[57] Rangel A, Camerer C, Montague PR (2008). A framework for studying the neurobiology of value-based decision making. Nat. Rev. Neurosci..

