# Supplementary material for: Sensorimotor strategy selection under time constraints in the presence of two motor targets with different values
Source: Sci Rep. 2021 Nov 15;11:22207. doi: 10.1038/s41598-021-01584-w (PMC8593016; doi:10.1038/s41598-021-01584-w)
Supplement: Supplementary file 1 — Supplementary Tables. [file 41598_2021_1584_MOESM1_ESM.pdf]

Supplementary information

Sensorimotor strategy selection under time constraints in the presence of two motor targets with different values

Ryoji Onagawa [1,2,3] and Kazutoshi Kudo[1]

1. Laboratory of Sports Sciences, Department of Life Sciences, Graduate School of Arts and Sciences, The University of Tokyo, Tokyo, Japan.
2. Research Fellow of Japan Society for the Promotion of Science, Tokyo, Japan.
3. Faculty of Science and Engineering, Waseda University, Tokyo, Japan

Table 1. Stats of Three-way repeated-measures ANOVA on |ΔIRD| (Fig. 4 in main text) *: P <.05, **: P <.01, ***: P <.001

Dependent variable	Independent variable	Main effect	Interaction	Simple main effects on num. of targets
**|Δ IRD|**	**time constraint [5]**	**number of targets:**	**time constraint × number of targets:**	**640< τ <880:** $P = 0.02$*
	**× number of targets [2]**	$F[1, 11] = 30.72$, $\eta_p^2 = 0.74$, $P < 0.001$***	$F[4, 44] = 4.22$, $\eta_p^2 = 0.28$, $P = 0.006$**	**880< τ <1120:** $P = 0.004$**
	**× target-separation angle [3]**	**target-separation angle:**	**number of targets × target-separation angle:**	**1120< τ <1360:** $P = 0.004$**
		$F[2, 22] = 556.6$, $\eta_p^2 = 0.98$, $P < 0.001$***	$F[2, 22] = 1.39$, $\eta_p^2 = 0.11$, $P = 0.269$	**1360< τ <1600:** $P < 0.001$***
		**time constraint:**	**time constraint × target-separation angle:**	
		$F[4, 44] = 0.617$, $\eta_p^2 = 0.053$, $P = 0.617$	$F[8, 88] = 0.954$, $\eta_p^2 = 0.08$, $P = 0.477$	
			**time constraint × number of targets × target-separation angle:**	
			$F[8, 88] = 0.523$, $\eta_p^2 = 0.08$, $P = 0.523$	

Table 2. Stats of two-way repeated measures ANOVA on movement pattern (Fig. 5 in main text) *: P <.05, **: P <.01, ***: P<.001

Dependent variable	Independent variables	main effect	interaction	Post Hoc Comparisons
**Freq. of Direct reaching**	**time constraint [5]**	**time constraint:**	$F[8, 88] = 1.44$, $\eta_p^2 = 0.12$, $P = 0.19$	**target-separation-Angle:** 30 vs 45: P=0.031*, 30 vs 60: P=0.008**, 45 vs 60: P = 0.458
	× **target-separation angle [3]**	$F[4, 44] = 7.26$, $\eta_p^2 = 0.40$, $P = 0.009$**		**time constraint:**
		**target-separation angle:**		**400< τ <640 vs 640< τ <880:** P=0.78, **400< τ <640 vs 880< τ <1120:** P = 0.559
		$F[2, 22] = 6.28$, $\eta_p^2 = 0.36$, $P = 0.024$*		**400< τ <640 vs 1120< τ <1360:** P=0.039*, **400< τ <640 vs 1360< τ <1600:** P < 0.001***
				**640< τ <880 vs 880< τ <1120:** P=0.589, **640< τ <880 vs 1120< τ <1360:** P = 0.069
				**640< τ <880 vs 1360< τ <1600:** P<0.001***, **880< τ <1120 vs 1120< τ <1360:** P = 0.557
				**880< τ <1120 vs 1360< τ <1600:** P=0.02*, **1120< τ <1360 vs 1360< τ <1600:** P = 0.557
**Freq. of Intermediate**	**time constraint [5]**	**time constraint:**	$F[8, 88] = 1.18$, $\eta_p^2 = 0.10$, $P = 0.33$	**target-separation-Angle:** 30 vs 45: P=0.031*, 30 vs 60: P=0.02*, 45 vs 60: P = 0.715
**behavior**	× **target-separation angle [3]**	$F[4, 44] = 5.40$, $\eta_p^2 = 0.33$, $P = 0.011$*		**time constraint:**
		**target-separation angle:**		**400< τ <640 vs 640< τ <880:** P=0.77, **400< τ <640 vs 880< τ <1120:** P = 0.218
		$F[2, 22] = 5.33$, $\eta_p^2 = 0.33$, $P = 0.027$*		**400< τ <640 vs 1120< τ <1360:** P=0.008**, **400< τ <640 vs 1360< τ <1600:** P = 0.003**
				**640< τ <880 vs 880< τ <1120:** P=0.777, **640< τ <880 vs 1120< τ <1360:** P = 0.141
				**640< τ <880 vs 1360< τ <1600:** P=0.061, **880< τ <1120 vs 1120< τ <1360:** P = 0.679
				**880< τ <1120 vs 1360< τ <1600:** P=0.409, **1120< τ <1360 vs 1360< τ <1600:** P = 0.777
**Freq. of Change-of-mind**	**time constraint [5]**	**time constraint:**	$F[8, 88] = 0.4$, $\eta_p^2 = 0.04$, $P = 0.748$	**time constraint:**
	× **target-separation angle [3]**	$F[4, 44] = 8.46$, $\eta_p^2 = 0.44$, $P = 0.002$**		**400< τ <640 vs 640< τ <880:** *P*=0.78, **400< τ <640 vs 880< τ <1120:** *P* = 0.559
		**target-separation angle:**		**400< τ <640 vs 1120< τ <1360:** *P*=0.039*, **400< τ <640 vs 1360< τ <1600:** *P* < 0.001***
		$F[2, 22] = 0.82$, $\eta_p^2 = 0.07$, $P = 0.455$		**640< τ <880 vs 880< τ <1120:** *P*=0.589, **640< τ <880 vs 1120< τ <1360:** *P* = 0.069
				**640< τ <880 vs 1360< τ <1600:** *P*<0.001***, **880< τ <1120 vs 1120< τ <1360:** *P* = 0.557
				**880< τ <1120 vs 1360< τ <1600:** *P*=0.02*, **1120< τ <1360 vs 1360< τ <1600:** *P* = 0.557

Table 3. Stats of one-way repeated measures ANOVA on movement pattern (Fig. 5 in main text) *: P <.05, **: P <.01, ***: P<.001

Dependent variable	Independent variable	main effect	Post Hoc Comparisons
**Freq. of Intermediate behavior**	**target-separation angle [3]**	$F[2, 22] = 5.828$, $\eta_p^2 = 0.346$, $P = 0.023$	**30 vs 45:** P = 0.023<br>**30 vs 60:** P = 0.015<br>**45 vs 60:** P = 0.711
**Freq. of Change-of-mind**	**target-separation angle [3]**	$F[2, 22] = 0.795$, $\eta_p^2 = 0.07$, $P = 0.464$	**30 vs 45:** P = 0.921<br>**30 vs 60:** P = 0.671<br>**45 vs 60:** P = 0.921

Table 4. Stats of One-sample t-test on $P_{high\text{-}value}$ (Fig. 7 in main text) *: P <.05, **: P <.01, ***: P <.001

Dependent variable	Test value	Results
**$P_{high\text{-}value}$ (400< τ <640)**	0.5	$t[11]=1.31$, d=0.38, P =0.219
**$P_{high\text{-}value}$ (640< τ <880)**	0.5	$t[11]=1.51$, d=0.44, P =0.16
**$P_{high\text{-}value}$ (880< τ <1120)**	0.5	$t[11]=5.15$, d=1.49, P <0.001***
**$P_{high\text{-}value}$ (1120< τ <1360)**	0.5	$t[11]=6.08$, d=1.75, P <0.001***
**$P_{high\text{-}value}$ (1360< τ <1600)**	0.5	$t[11]=6.57$, d=1.90, P <0.001***

Table 5. Stats of two-way repeated measures ANOVA on performance indexes (Fig. 7 in main text) *: P <.05, **: P <.01, ***: P<.001

Dependent variable	Independent variables	main effect	interaction	Simple main effects on time constraints	Simple main effects on num. of targets
**RT (reaction time)**	**time constraint [5]**	**time constraint:** $F[4, 44] = 42.79$, $\eta_p^2 = 0.80$, $P < 0.001$***	$F[4, 44] = 25.63$, $\eta_p^2 = 0.70$, $P < 0.001$***	**double-targets condition:** P-values < 0.001***	**all levels:** P-values < 0.001***
	**× number of targets [2]**	**number of targets:** $F[1, 11] = 77.13$, $\eta_p^2 = 0.86$, $P < 0.001$***		**single-target condition:** P-values < 0.001***	
**IMV (initial movement**	**time constraint [5]**	**time constraint:** $F[4, 44] = 41.53$, $\eta_p^2 = 0.80$, $P < 0.001$***	$F[4, 44] = 1.30$, $\eta_p^2 = 0.11$, $P = 0.29$		
**velocity)**	**× number of targets [2]**	**number of targets:** $F[1, 11] = 0.12$, $\eta_p^2 = 0.011$, $P = 0.74$			
**MT (movement time)**	**time constraint [5]**	**time constraint:** $F[4, 44] = 2.63$, $\eta_p^2 = 0.19$, $P = 0.047$*	$F[4, 44] = 2.63$, $\eta_p^2 = 0.19$, $P = 0.047$*	**double-targets condition:** P-values < 0.001***	**all levels:** P-values > 0.05
	**× number of targets [2]**	**number of targets:** $F[1, 11] = 0.70$, $\eta_p^2 = 0.057$, $P = 0.43$		**single-target condition:** P-values < 0.001***	
**PL (path lengths)**	**time constraint [5]**	**time constraint:** $F[4, 44] = 7.02$, $\eta_p^2 = 0.057$, $P < 0.001$***	$F[4, 44] = 6.49$, $\eta_p^2 = 0.37$, $P = 0.006$**		**880< τ <1120:** $P = 0.042$*
	**× number of targets [2]**	**number of targets:** $F[1, 11] = 11.90$, $\eta_p^2 = 0.52$, $P = 0.005$**			**1120< τ <1360:** $P = 0.001$**
					**1360< τ <1600:** $P = 0.005$**
**mean score**	**time constraint [5]**	**time constraint:** $F[4, 44] = 62.68$, $\eta_p^2 = 0.85$, $P < 0.001$***	$F[4, 44] = 7.27$, $\eta_p^2 = 0.40$, $P = 0.002$**		**640< τ <880:** $P = 0.002$**
	**× number of targets [2]**	**number of targets:** $F[1, 11] = 0.004$, $\eta_p^2 = 3.59 \times 10^{-4}$, $P = 0.952$			**880< τ <1120:** $P = 0.016$*
**$P_{temporal}$**	**time constraint [5]**	**time constraint:** $F[4, 44] = 51.71$, $\eta_p^2 = 0.83$, $P < 0.001$***	$F[4, 44] = 6.28$, $\eta_p^2 = 0.36$, $P < 0.009$**		**640< τ <880:** $P = 0.001$**
	**× number of targets [2]**	**number of targets:** $F[1, 11] = 17.25$, $\eta_p^2 = 0.61$, $P = 0.002$**			**880< τ <1120:** $P = 0.017$*
**$P_{spatial}$**	**time constraint [5]**	**time constraint:** $F[4, 44] = 8.77$, $\eta_p^2 = 0.44$, $P < 0.001$ ***	$F[4, 44] = 0.50$, $\eta_p^2 = 0.043$, $P = 0.74$		
	**× number of targets [2]**	**number of targets :**$F[1, 11] = 6.47$, $\eta_p^2 = 0.37$, $P = 0.027$*			
